# Community-acquired Methicillin-resistant *Staphylococcus aureus* in Children, Taiwan

**DOI:** 10.3201/eid1208.051570

**Published:** 2006-08

**Authors:** Wen-Tsung Lo, Wei-Jen Lin, Min-Hua Tseng, Sheng-Ru Wang, Mong-Ling Chu, Chih-Chien Wang

**Affiliations:** * National Defense Medical Center, Taipei, Taiwan, Republic of China;; † Tri-Service General Hospital, Taipei, Taiwan, Republic of China

**Keywords:** Panton-Valentine leukocidin, community-acquired methicillin-resistant Staphylococcus aureus, nasal colonization, Taiwan, dispatch

## Abstract

Highly virulent community-acquired methicillin-resistant *Staphylococcus aureus* (CA-MRSA) with Panton-Valentine leukocidin (PVL) is common worldwide. Using antimicrobial drug susceptibility testing, staphylococcal cassette chromosome *mec* typing, exotoxin profiling, and pulsed-field gel electrophoresis typing, we provide evidence that supports the relationship between nasal strains of PVL-positive MRSA and community-acquired disease.

Panton-Valentine leukocidin (PVL) is a 2-component cytotoxin that targets human and rabbit polymorphonuclear cells, monocytes, and macrophages (*1*). Gene products of PVL (lukS-PV and lukF-PV), which are encoded by contiguously located, cotranscribed genes (*lukS-PV* and *lukF-PV*), assemble as hetero-oligomers and synergistically exert cytolytic pore-forming activity (*1*). PVL is mainly associated with primary cutaneous infections, especially furuncles, and with severe necrotizing community-acquired pneumonia (*2*). The PVL locus is present in most community-acquired methicillin-resistant *Staphylococcus aureus* (CA-MRSA) isolates studied and is a stable marker of CA-MRSA strains worldwide (*3*).

In a previous study, we found that CA-MRSA skin and soft tissue infections among Taiwanese children are caused by a predominantly endemic strain that has PVL genes (*4*). Nasal carriage of MRSA also plays a key role in the epidemiology and pathogenesis of community-associated disease (*5*). Therefore, our prospective investigation sought to 1) determine the prevalence of PVL-positive *S*. *aureus* among isolates from children with various staphylococcal diseases and asymptomatic nasal colonization, and 2) test the hypothesis that CA-MRSA infection is associated with community PVL-positive MRSA nasal carriage.

## The Study

The study protocol was reviewed and approved by the National Defense Medical Center Institutional Review Board. Two collections of *S*. *aureus* isolates were used. A list of all children <14 years of age hospitalized with various staphylococcal infections during the period from December 2003 to November 2005 was compiled from records at the clinical microbiology laboratory at the Tri-Service General Hospital in Taipei. The first collection of 144 infecting strains was further categorized into 11 types of staphylococcal infection on the basis of the clinical details provided. A case was considered community acquired if MRSA was isolated from cultures of specimens obtained within 72 hours after hospitalization. Risk factors for MRSA infection included hospitalization <12 months before the date of MRSA isolation; history of any surgical procedure; history of endotracheal intubation; underlying chronic disorder; antimicrobial drug therapy <12 months before the date of MRSA isolation; presence of an indwelling venous or urinary catheter; or household contact with a person with an identified risk factor or a worker in a healthcare environment (*6*).

A second collection of 300 colonizing strains was obtained during the same period by culturing samples from the anterior nares of 1,195 healthy children in the community. Eligible participants were <14 years of age with no acute medical problem who either visited a healthcare facility for a well-child checkup or attended 1 of 7 kindergartens in Taipei.

MRSA identification and antimicrobial drug susceptibility were determined according to the Clinical Laboratory Standards Institute (formerly known as the National Committee for Clinical Laboratory Standards) guidelines (*7**,**8*). Staphylococcal cassette chromosome *mec* (SCC*mec*) elements were typed and PVL genes were detected as described (*2**,**9**,**10*). Sequences specific for *sea* to *see*, *seg* to *sei*, *eta*, *etb*, and *tst*, which encode staphylococcal enterotoxins (SEA to SEE, and SEG to SEI), exfoliative toxins (ETA and ETB), and toxic shock syndrome toxin-1, respectively, were detected by using methods described by Jarraud et al. (*11*). Pulsed-field gel electrophoresis (PFGE) was performed by using a CHEF Mapper XA system (Bio-Rad Laboratories, Hercules, CA, USA) according to a published protocol (*12*).

Data were analyzed by the Mantel-Haenszel test and χ^2^ test with SPSS version 10.0 software (SPSS, Chicago, IL, USA). A p value <0.05 was considered significant.

Of the 444 isolates examined, PVL-positive isolates constituted 23% of all *S*. *aureus* isolates analyzed (Table 1). Among 144 isolates (67 methicillin-susceptible *S*. *aureus* isolates and 77 MRSA isolates) from different staphylococcal infections, 82 (56.9%) were PVL positive, and most were associated with skin and soft tissue infections (especially furuncles). In contrast, only 18 (6%) of 300 colonizing isolates had the PVL locus. PVL genes were also found in isolates associated with other deep-space infections including pyomyositis, osteomyelitis, and septic arthritis. Of 1,195 healthy children who were screened, 89 (7.4%) had cultures with MRSA. Only 15 (16.9%) of 89 community MRSA-colonizing strains were PVL positive.

**Table 1:** Association of Panton-Valentine leukocidin-positive *Staphylococcus aureus* isolates with types of staphylococcal infection and colonization*

Origin of sample	No. isolates	No. (%) PVL-positive isolates	Risk ratio (95% CI)†	p value‡
Furuncles	7	7 (100)	8.000 (1.279–50.040)	0.001
Abscess	9	8 (89)	7.111 (1.121–45.129)	0.002
Carbuncle	26	20 (77)	6.154 (0.972–38.959)	0.001
Cellulitis	25	19 (76)	6.080 (0.959–38.535)	0.002
Staphylococcal scarlet fever	27	17 (63)	5.037 (0.787–32.229)	0.013
Wounds§	20	5 (25)	2.000 (0.275–14.548)	0.475
Pyoderma	5	1 (20)	1.600 (0.127–20.219)	0.726
Pneumonia¶	8	1 (13)		
Bullous impetigo	6	0	NA/NM	NA/NM
Bacteremia	7	0	NA/NM	NA/NM
Other invasive infection#	4	4 (100)	8.000 (1.279–50.040)	0.006
Colonization	300	18 (6)	0.480 (0.073–3.169)	0.452
Total	444	100 (23)		

*PVL, Panton-Valentine leukocidin; CI, confidence interval; NA, not applicable; NM, not measured. †Risk ratio is the ratio of the risk of being PVL positive in the presence of a particular type of infection or colonization to the absence of that type of infection or colonization. ‡By Mantel-Haenszel test. §Mostly postsurgical. ¶Reference group for statistical analysis. #Includes pyomyositis, osteomyelitis, and septic arthritis.

Among the 144 *S*. *aureus* isolates obtained from various types of staphylococcal disease, 32 (22.2%) isolates were further confirmed as CA-MRSA according to the inclusion criteria (abscess [n = 5], carbuncles [n = 6], cellulitis [n = 9], furuncles [n = 3], pyomyositis [n = 1], osteomyelitis [n = 1], pneumonia [n = 1], and staphylococcal scarlet fever [n = 6]); all contained the genes encoding PVL. Antibiograms of 15 PVL-positive MRSA-colonizing strains did not differ significantly from those of 32 CA-MRSA–infecting strains with respect to clindamycin, erythromycin, gentamicin, and chloramphenicol. All 47 PVL-positive MRSA isolates were resistant to penicillin G, and none had reduced susceptibility to vancomycin, teicoplanin, trimethoprim-sulfamethoxazole, fusidic acid, or ciprofloxacin.

To gain insight into the association between nasal strains of PVL-positive MRSA and community-acquired disease, we designed a comparative study in which the colonization and clinical samples were collected during the same period. Results of SCC*mec* typing and exotoxin profiling for both community MRSA-colonizing strains and CA-MRSA–infecting strains are shown in Table 2. Of 74 colonizing strains that did not have the PVL locus, most (94.6%) had SCC*mec* IV, and only 1 (1.35%) had SCC*mec* V_T_. Conversely, irrespective of origin, PVL-positive MRSA strains were more likely to have SCC*mec* V_T_ than were the PVL-negative MRSA-colonizing strains (p<0.001). Regarding the exotoxin profiles, the most frequently encoded toxin gene among PVL-positive MRSA isolates was *seb* (97.9%). PVL-positive MRSA-colonizing strains and CA-MRSA–infecting strains were more likely to have genes that encoded SEB than were PVL-negative CA-MRSA–colonizing strains (p = 0.006). Genes for SEG/SEI were found only in PVL-negative MRSA-colonizing strains (p<0.001).

**Table 2:** Distribution of staphylococcal cassette chromosome (SCC)*mec* types and exotoxin patterns among methicillin-resistant *Staphylococcus aureus* (MRSA) strains collected from community-acquired (CA) MRSA infections and nares cultures*

	CA-MRSA–colonizing strains		CA-MRSA–infecting strains†
Characteristic	PVL positive (n = 15)	PVL negative (n = 74)	(n = 32)
SCC*mec* type, no. (%) of isolates			
II	0	1 (1.35)	0
III	0	1 (1.35)	0
IIIA	0	1 (1.35)	0
IV	7 (46.7)	70 (94.6)‡	5 (15.6)
V_T_§	8 (53.3)	1 (1.35)‡	27 (84.4)
No. (%) of isolates positive for production of other toxins			
ETA	0	2 (2.7)	0
ETB	1 (6.7)	2 (2.7)	0
TSST-1	0	5 (6.6)	1 (3.1)
SEA	1 (6.7)	6 (8.1)	0
SEB	14 (93.3)	57 (77.0)¶	32 (100)
SEC	0	4 (5.4)	0
SED	0	0	0
SEE	0	1 (1.4)	0
SEG/SEI	0	30 (40.5)‡	0
SEH	0	1 (1.4)	0

*PVL, Panton-Valentine leukocidin; ETA, exfoliative toxin A; ETB, exfoliative toxin B; TSST-1, toxic shock syndrome toxin-1; SEA, staphylococcal enterotoxin A; SEB, staphylococcal enterotoxin B; SEC, staphylococcal enterotoxin C; SED, staphylococcal enterotoxin D; SEE, staphylococcal enterotoxin E; SEG, staphylococcal enterotoxin G; SEI, staphylococcal enterotoxin I; SEH, staphylococcal enterotoxin H. †All 32 CA-MRSA–infecting strains were PVL positive. ‡p<0.001 by χ^2^ test for PVL-positive MRSA-colonizing strains and CA-MRSA–infecting strains vs. PVL-negative MRSA-colonizing strains. §V_T_ refers to the SCC*mec* V_T_ element in strain TSGH 17 from Taiwan (*10*). ¶p = 0.006 by χ^2^ test for PVL-positive MRSA-colonizing strains and CA-MRSA–infecting strains vs. PVL-negative MRSA-colonizing strains.

Diverse pulsotypes were found among the 47 PVL-positive MRSA strains subjected to PFGE typing (Figure). Two clusters that included 33 (70.2%) isolates were distinguished at the 70% similarity level. None of the isolates were linked on review of epidemiologic data derived from medical records. Except for 1 colonizing isolate (C7) and 2 infecting isolates (I22 and I23) that carried SCC*mec* IV, the other 30 (90.9%) isolates from clusters I and II had SCC*mec* V_T_.

**Figure:**
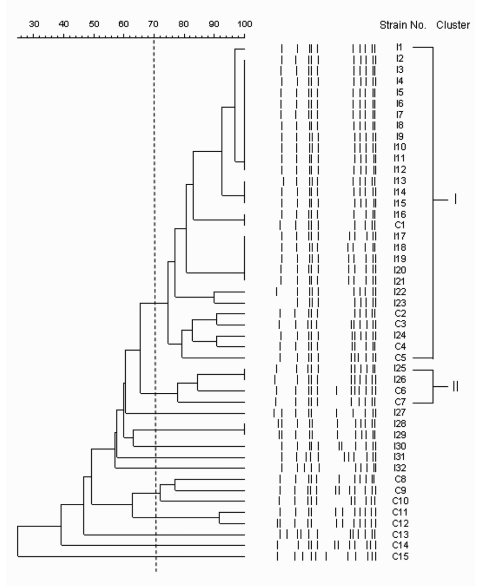
Pulsed-field gel electrophoresis patterns and phylogenetic tree of 47 methicillin-resistant *Staphylococcus aureus* (MRSA) isolates with Panton-Valentine leukocidin (PVL) genes. Banding patterns were digitalized and analyzed with Molecular Analyst Fingerprinting, Fingerprinting Plus, and Fingerprinting DST software (Bio-Rad Laboratories, Hercules, CA, USA). The grouping method was performed to deduce a dendrogram from the matrix by the unweighted pair group method with arithmetic averages clustering technique after calculation of similarities using the Pearson correlation coefficient between each pair of organisms. The scale indicates the level of pattern similarity. Similarities >70% represent clonal spread of strains. The first letter of each isolate designation indicates the type of the isolate as follows: I, community-acquired MRSA-infecting isolates; C, PVL-positive MRSA-colonizing isolates.

## Conclusions

This is the first epidemiologic study of PVL-positive *S*. *aureus* in Taiwan. The prevalence of PVL-positive *S*. *aureus* among isolates collected from various types of staphylococcal infections was 56.9%; previous surveys reported rates of <5% to 12.4% (*13**–**15*). This higher prevalence is probably related to the greater proportion of pediatric patients with cutaneous infections. The frequencies of infections associated with these organisms were similar to those in previous studies (*2**,**13**,**14*). Overall, the outcomes of infections with PVL-positive strains were excellent and comparable to that of PVL-negative strains. Only 1 death occurred.

Four factors that support the relationship between nasal strains of PVL-positive MRSA and community-acquired disease. First, both PVL-positive MRSA-colonizing strains and CA-MRSA–infecting strains had consistent antibiograms. Second, most PVL-positive MRSA-colonizing strains and CA-MRSA–infecting strains had SCC*mec* V_T_, whereas a high prevalence of SCC*mec* IV was found among PVL-negative MRSA-colonizing strains. Third, PVL-positive MRSA-colonizing strains and CA-MRSA–infecting strains had consistent exotoxin profiles, which differed from those of PVL-negative MRSA-colonizing strains. Fourth, PFGE findings indicate that some PVL-positive MRSA isolates that colonized children who remained asymptomatic were clonally related to clinically isolated CA-MRSA–infecting strains, especially those with SCC*mec* V_T_, compared with SCC*mec* IV (6/8, 75% and 1/7, 14.3%, respectively).

Several study limitations merit consideration. First, our study is a snapshot in time because the molecular epidemiology of CA-MRSA is constantly changing. Second, we were unable to determine risk for infection because children colonized with community PVL-positive MRSA were not followed-up longitudinally. Finally, our results are geographically distinct and may not be generalized to the global population.

Our study showed that PVL genes are carried by a large number of *S*. *aureus* isolates, especially among those causing disease. We provide evidence that links community PVL-positive MRSA-colonizing strains to CA-MRSA–infecting strains from various types of staphylococcal infection.
